# Accidental Ingestion of Endodontic File: A Case Report

**DOI:** 10.1155/2012/278134

**Published:** 2012-04-17

**Authors:** Hrushikesh P. Saraf, Pradnya P. Nikhade, Manoj G. Chandak

**Affiliations:** Department of Conservative Dentistry and Endodontics, SPDC, Sawangi, 442004 Wardha, India

## Abstract

Ingestion of the endodontic instrument during root canal treatment is rare but can result in serious complications. The present paper reports a case in which endodontic file was accidentally swallowed by the patient undergoing root canal therapy, which entered digestive tract and passed uneventfully.

## 1. Introduction

Ingestion of foreign body is a common clinical problem in children. Ingestion still occurs in adults but is most of the times accidental or in psychiatric patients. In dental operatory, the ingested foreign body may include teeth, restorations, restorative materials, instruments, rubber dam clamps, gauze packs, and so forth [1–4]. Grossman [5] determined that 87% of the ingested foreign bodies entered the gastrointestinal tract, and 13% entered the respiratory tract. Most of the foreign bodies that entered the gastrointestinal tract pass spontaneously. Only 10–20% cases require nonsurgical intervention, and 1% or less requires surgical removal [6]. This paper discusses a case report of accidental ingestion of endodontic file and its management.

## 2. Case Report

A 38-years-old female reported to the Department of Conservative Dentistry and Endodontics with the chief complaint of pain in mandibular left first molar tooth. On clinical examination, mandibular left first molar was found carious. On radiographic evaluation caries with mandibular, left first molar was seen involving pulp, so routine root canal treatment was undertaken. Access opening was done under rubber dam application. Patient was having extreme salivation, coughing and gagging reflex. While taking the radiograph for working length, frame was removed as it was rigid metallic frame. The patient suddenly got gagging reflex and then coughed and moved. During coughing the 15-number K file which was snugly fitting in the root canal slipped on the floor of mouth and was swallowed by patient unknowingly. The patient complained of excessive gagging with the sensation of something sticking in her throat. Patient was instructed to cough forcefully, but file could not be retrieved. Thorough examination was done using tongue depressor but was not productive. There was no evidence of airway compromise, respiratory distress, or abdominal tenderness. Patient was informed about the accident and was assured.

**Figure 1:**
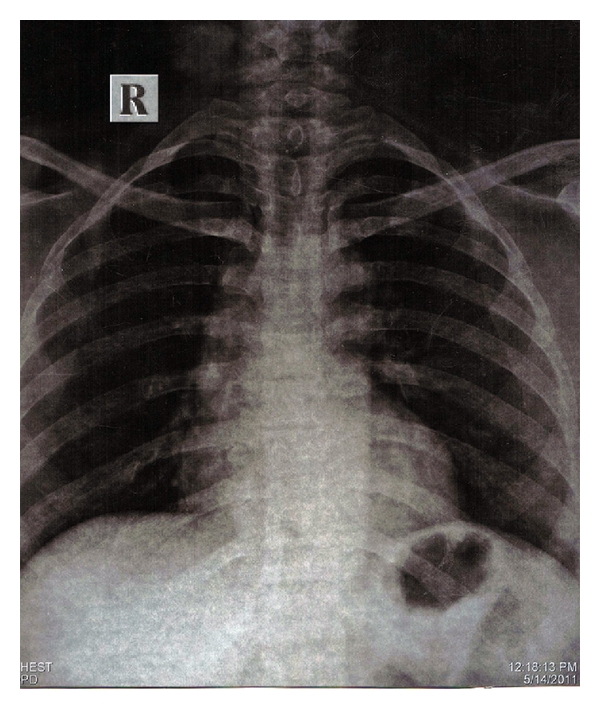
Chest radiograph after half an hour, showing no evidence of instrument.

**Figure 2:**
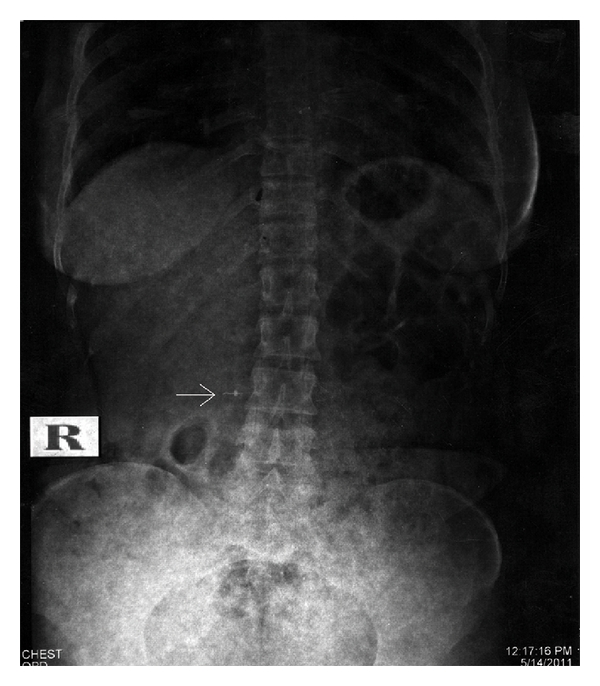
Abdomen radiograph after half an hour, showing instrument in lumbosaccaral region of intestine.

**Figure 3:**
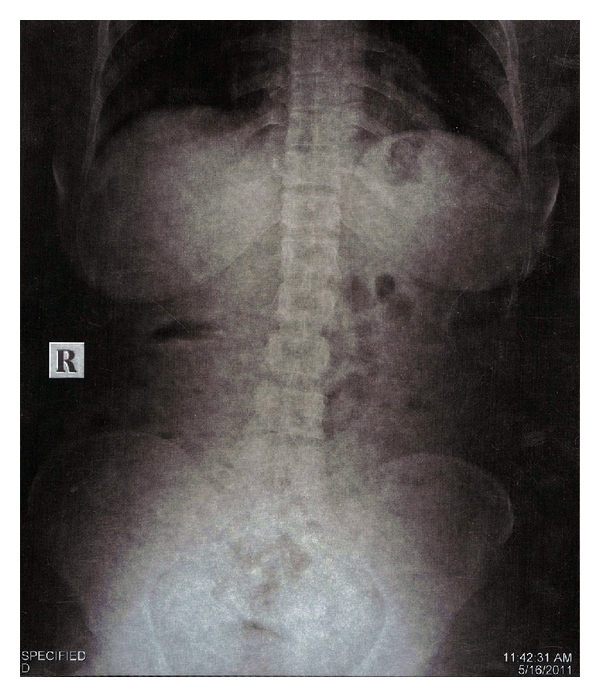
Abdominal radiograph after 3 days with no evidence of instrument.

Patient was taken to Radiology Department, a chest and an abdominal radiographs were taken. Chest radiograph was clear (Figure 1). The file was detected in abdominal radiograph (Figure 2), suggesting its presence in lower gastrointestinal tract at lumbosacral level, which was confirmed by a radiologist. The patient was informed and reassured. A diet high in roughage was prescribed to aid in the passage of instrument through intestinal tract. She was warned of the possible symptoms, that might indicate a perforation of the intestine and was told to examine her stools for file at every bowel movement. Patient was kept under close observation. Her progress was followed by abdominal radiograph on the 3rd day. Meanwhile, patient had no symptoms like blood in stools, and abdominal tenderness. The abdominal radiograph (Figure 3) was clear with no evidence of file, suggested that that the file passed out which was confirmed by radiologist. Patient was reassured, and root canal treatment was completed.

## 3. Discussion

Ingested foreign bodies that lodge into gastrointestinal tract pass through the gastrointestinal tract within a few days to a month [7]. When such cases are not diagnosed or treated appropriately, it may cause serious complications. Owing to the shape and sharpness of the instrument, there are chances of perforation. Once the instrument is lost in the oropharynx, it is very important to determine whether the instrument has entered the digestive tract or respiratory tract. Radiographic examination with posteroanterior and lateral chest radiograph, abdominal radiograph is mandatory for determining the location, size, and nature of foreign body. In the reported case chest and abdomen, radiographs were advised. In this cases chest radiograph was advised as patient was complaining of something sticking in throat. In case of foreign body that is radiolucent, other diagnostic methods includes computed tomography, magnetic resonance imaging, and endoscopy. Ninety percent of the ingested foreign bodies pass through the gastrointestinal tract uneventfully. Endodontic file has been previously reported to pass out through the gastrointestinal system within 3 days without incident [8]. In this case also the ingested file passed out through gastrointestinal tract without any symptoms. Careful monitoring with radiographic evaluation and high fibre diet is generally the preferred management protocol [9]. If the foreign body that has passed into the stomach and is less than 6 cm in length and 2 cm in diameter, there is 90% chance of passage through pylorus and ileocaecal valve [10]. With sharp object, the most common sites of perforation are the lower oesophagus and terminal ileum [10]. Abdominal pain and/or a positive stool occult blood test may indicate signs of intestinal perforation, impaction, or obstruction; medical or surgical intervention for removal is required in such cases.

Inflammatory bowel disease, tumours, diverticula, hernias, adhesions, anatomic narrowing, or acute angulations of the alimentary canals also increase the risk of perforation (Lyons II and Tsuchida 1993) [11]. Fortunately, the present patient had good general health with no history of bowel diseases.

Entry of a foreign body to the respiratory tract is potentially life-threatening, and the object requires prompt removal [12]. Vigorous and spasmodic cough and difficulty in breathing frequently occur immediately; however, a period without symptoms can last for years. The most common signs and symptoms of foreign body aspiration include coughing, wheezing, and decreased breathing sounds. Foreign bodies tend to be lodged preferentially in the right bronchial tree because of its anatomical vertical position [12].

 Ingestion or aspiration of foreign bodies can be easily prevented by the universal use of rubber dam isolation (Cohen and Schwartz 1987). Flexible rubber dam frames are available, which can facilitate radiographs during treatment without removal of frame. It offers effective protection against aspiration or swallowing of endodontic instruments, broken burs, restorative materials, and pins. While the rubber dam reduces the risk of aspiration during restorative procedures, it is possible for the dam clamp itself to be aspirated. To reduce this risk, dental floss should be tied to secure rubber dam clamp [13]. Dental floss can also be used to tie the endodontic files. Electronic apex locators can also be useful for working length determination avoiding rubber dam frame removal. Many dental techniques preclude the use of the rubber dam, particularly during routine oral surgery and prosthodontic procedures. An alternative is to place a 4 × 4-inch gauze protective barrier in the oral cavity distal to the area, where small items are being manipulated. The dentist may also prevent cast restoration being aspirated by using dental floss. Dentist should also instruct patients that if an object falls on the tongue, they should try to suppress the swallowing reflex and turn their heads to the side. An impression procedure may put a patient at a risk of aspirating the impression material if a large amount of material and/or low viscosity material is introduced to the posterior oral cavity. Therefore, use of the most viscous material available that will achieve the desired level of accuracy for the impression procedure is recommended [14].

Strategies to prevent aspiration of foreign bodies:

- use a rubber dam with flexible frames;
- endodontic files can be tied with floss to prevent ingestion;
- use of electronic apex locators and rotary instrument can help preventing file ingestion;
- use a gauze throat pack;
- use high-velocity evacuation;
- use Washfield technique;
- use a high-viscosity type of impression material;
- use a custom tray, with an open palate design for maxillary arch impression;
- observe the entire impression procedure;
- use a more upright position if possible;
- provide thorough instructions to the patients.

## 4. Conclusion

Handling of dental objects requires particular care, especially where the patient is supine or semirecumbent. Dentist should be able to manage an emergency situation, in which patient accidentally swallow dental instrument.
